# Forest neighbourhoods and healthcare access for Adivasi communities in India: A critical interpretive synthesis

**DOI:** 10.36368/jcsh.v2i2.1187

**Published:** 2025-11-05

**Authors:** Anika Juneja, NS Prashanth, Surekha Garimella, Anna-Karin Hurtig

**Affiliations:** 1 Institute of Public Health, Bengaluru, India; 2 The George Institute of Global Health, India; 3 Department Epidemiology and Global health, Umeå university, Sweden

**Keywords:** Forest, neighbourhood, Adivasi, access, social, environment, Indigenous, Bosque, vecindario, Adivasi, acceso, social, entorno, Indígenas

## Abstract

**Introduction:**

Environments where people live and work shape resources and opportunities available to them and studying healthcare access in relation to people’s living environments helps in understanding structural factors beyond individual factors. This is especially relevant for many Adivasi communities whose lives are closely connected with forests.

**Methods:**

We used the critical interpretive synthesis method, a flexible, critical and iterative approach to literature synthesis. We conceptualised health and healthcare access in relation to neighbourhood environment and used this lens to examine healthcare access in Adivasi communities living in forest neighbourhoods in India.

**Results:**

We developed a lens of neighbourhood as a physical and social environment and used it to build a conceptual framework describing forest neighbourhoods in India. We describe forest neighbourhoods in terms of their built and social environment. The availability of mobile networks, condition of roads, flooding of streams during rains and the forms of transport available constitute the built environment. There are two important components of the social environment, first is the connection of the Adivasi people with the forest and second is the institutional environment comprising of different actors working in the forest neighbourhood. The life of Adivasi people is connected with the forest through their livelihood, nutrition, physical and mental well-being and their ecological knowledge about the forest from their lived experience. The institutional environment consists of different actors that shape the built and social environment that comprise the government institutions, private for-profit providers, civil society organisations, traditional healers and the forest department.

**Conclusion:**

While working on Adivasi health, it is critical to consider their connection with the forest. Exploring forest neighbourhoods as physical and social environments can help examine distribution of public services and how they are shaped by external policies and actors working in the neighbourhood. This could shift the focus of Adivasi health and healthcare interventions away from the current emphasis on individual-level health interventions.

## Abstract (es)

**Introducción:**

Los entornos donde las personas viven y trabajan moldean los recursos y oportunidades disponibles para ellas, y estudiar el acceso a la atención médica en relación con estos entornos ayuda a comprender factores estructurales más allá de los individuales. Esto es especialmente relevante para muchas comunidades Adivasi, cuyas vidas están estrechamente ligadas al bosque.

**Métodos:**

Utilizamos el método de síntesis interpretativa crítica, un enfoque flexible, crítico e iterativo para la síntesis de literatura. Conceptualizamos la salud y el acceso a la atención sanitaria en relación con el entorno vecinal, y empleamos este enfoque para analizar el acceso a la atención médica en comunidades Adivasi que habitan en zonas boscosas de la India.

**Resultados:**

Desarrollamos una perspectiva del vecindario como entorno físico y social, y la utilizamos para construir un marco conceptual que describe los vecindarios forestales en la India. Los describimos en términos de su entorno construido y social. La disponibilidad de redes móviles, el estado de las carreteras, las inundaciones de arroyos durante la temporada de lluvias y los medios de transporte disponibles constituyen el entorno construido. Hay dos componentes clave del entorno social: el primero es la conexión del pueblo Adivasi con el bosque, y el segundo es el entorno institucional, compuesto por diversos actores que operan en el vecindario forestal. La vida del pueblo Adivasi está conectada con el bosque a través de su sustento, alimentación, bienestar físico y mental, y conocimiento ecológico adquirido de su experiencia vivida. El entorno institucional incluye actores que moldean tanto el entorno construido como el social: instituciones gubernamentales, proveedores privados con fines de lucro, organizaciones de la sociedad civil, curanderos tradicionales y el departamento forestal.

**Conclusión:**

Al trabajar en temas de salud con los Adivasi, es fundamental considerar la conexión entre su vida y el bosque. Explorar los vecindarios forestales como entornos físicos y sociales permite examinar cómo se distribuyen los servicios públicos y cómo estos son moldeados por políticas externas y actores presentes en el vecindario. Esto podría cambiar el enfoque de las intervenciones en salud y atención sanitaria Adivasi, alejándolo de la actual orientación centrada en el individuo.

## Introduction

Over 104 million tribal people live in India, and they account for 8.6% of the country’s population. Almost 90% of the tribal population in India lives in rural areas including hilly and forested areas. They are categorised as those living in Schedule 5 areas in central India, Schedule 6 areas in northeast India and those living outside Schedule areas. Schedule areas were created under Article 244 of the Constitution with special governance mechanisms to uphold tribal autonomy and culture in order to secure social, political and economic justice for these communities. Provision of Panchayats Act which is an Act of Parliament to set up local governments, was brought in 1996 to empower *Gram Sabhas* which is a village level legislative body that promotes development and democracy. A central Ministry of Tribal Affairs was created in 1999 to ensure socio-economic development of Scheduled Tribes [1]. Different terms are used inter-changeably for Adivasi communities in India such as tribal, Adivasi and Scheduled Tribe but each term has a different history of its origin. The term scheduled tribe (ST) is a political and administrative term which was used by the anthropological survey of India in when 461 tribal communities were identified. The term Adivasi which is the Indian version of the term ‘indigenous’, originally was used to describe original inhabitants of an area, however over time the meaning of the term has shifted towards belonging to communities that face challenges in entitlement over their land, forest, river and related resources [2].

In a report by Thresia et al. [3], almost half of the people that were displaced in South Asia due to creation of dams, mines and industries were from the Scheduled Tribes (ST). Adivasi communities have traditionally engaged with livelihoods such as collection of forest produce, shifting cultivation, hunting gathering and artisans. As per a report by an expert committee on tribal health, 40.6% of tribal people are affected by poverty and do not have access to literacy [1]. The same report attributes gaps in health infrastructure and resources to geographic and socio-economic challenges. As per the same report, more than half of the tribal people have been dependent on government healthcare facilities for outpatient and inpatient treatment. For many of the Adivasi people, traditional healers are their first point of contact as they are easily accessible and offer spiritually safe care, which many times is lacking in the modern healthcare system.

Individual behaviours play out in specific geospatial and social environments and thus it is important to explore behaviours of individuals in relation to their environments. While many public health interventions focus on behaviour change of individuals, exploring the environment in which behaviours takes place, can help understand and build environment focused solutions [4]. Environments where people live, work, play and grow are key parts of social determinants of health and significantly shape the resources and opportunities available to them [5]. Income, occupation and education are often attributed to individuals but they are also determined by the place one lives in and the opportunities available there [6]. Roads and public transport help people reach healthcare facilities, schools and facilitate health workers and other services reach them, especially in times of an emergency. Policies, institutions, organisations and processes govern distribution of healthcare services in a neighbourhood and these are often outside of individual control [7]. While interacting with the healthcare system, the built environment such as roads and transport and the social environment such as social networks and collective efficacy can play a role in supporting or interfering with this interaction. [8]

For many Adivasi communities in India, forests are a critical part of their living environments as their identity, livelihood, nutrition and well-being is deeply connected with the forest. Some of the Adivasi communities are no longer staying in and around forests and have relocated to other rural and urban areas. For this study, we focus on the Adivasi communities living in and around forests in India. When we talk about healthcare access for forest-associated Adivasi communities, it is important to examine their healthcare access in relation to their living environments. For this study we use the term neighbourhood as we think it to be appropriate to examine healthcare access in relation to the living environment. Neighbourhoods are not just physical places where people live but are also defined by availability of different services that people would use as part of life, including health-related resources. Neighbourhood has been defined as “a place with a unique system of health relevant resources and social relationships embedded within geographical borders” [9]. It is defined as “a relatively granular geographic area surrounding the individual’s place of residence and containing physical, social and economic attributes with which the individual interacts in daily activities of travel, work, recreation and accessing resources” [8].

### Literature on neighbourhoods and health

A study by Bell et al. in Canada [10] that focussed on spatial and aspatial characteristics of neighbourhood in relation to healthcare access, reported that health outcomes such as low birth weight, infant mortality and cardio-vascular disease and behaviours such as smoking, alcohol consumption, diet and physical activity are impacted by neighbourhood residence. Availability of services in the neighbourhoods such as parks, grocery stores, alcohol and fast-food outlets impact these behaviours. Studies based in United States report higher levels of physical activity, better cardiorespiratory fitness and greater social capital in people who live in more walkable neighbourhoods [8]. Neighbourhood organisations such as schools, civil society organisations and religious places act as places of social networks where information and social support, including that related to healthcare, may be obtained [11].

According to Ryvicker [8], the term-built environment includes structures such as roads, highways, parks, playgrounds, public transportation and a mix of residential and commercial use of land. The study explores healthcare navigation in relation to the built environment and how these structures influence individual behaviour and the ways in which people travel, socialize, work, recreate, interact with the economy and utilize social and health services. It also shows that an individual’s ability to use locally available healthcare resources is affected by access to public or private transport, availability of social support and walkability of the neighbourhood.

The previously mentioned study by Bell et al. [10] mapped spatial and aspatial characteristics in access to healthcare, aspatial characteristics referring to social characteristics and accessibility to be considerably reduced for linguistic minorities in Canada. According to the study, social ties and networks, safety, perception of crime and socioeconomic status are important neighbourhood social attributes. In another study from United States, overall physical activity levels in older adults, were associated with people’s perceptions of neighbourhood safety and social cohesion, more than that of the built environment characteristics. Most of these studies come from western parts of the world such as United States and Canada and there are very few studies from India about neighbourhood environment and healthcare access [8].

## Methods

We use critical interpretive synthesis (CIS) as a method of literature synthesis focusing on theorizing and building conceptual frameworks using a critical lens [12]. This method is a flexible, iterative, critical and interpretive approach to literature synthesis [13]. We critically examine the local environment, not only in terms of its physical features but also its social features to build a comprehensive understanding of place and use it to examine healthcare access in forest living Adivasi communities in India.

There are two review questions for the synthesis:

i) How is the neighbourhood environment conceptualised in relation to healthcare access?
ii) How do neighbourhood environments shape healthcare access for Indian Adivasi communities living in forest neighbourhoods?

This literature synthesis is part of the doctoral work of the first author who is a PhD student associated with the Centre for training research and innovation in tribal health (CTRITH). This Centre is a learning site engaging in participatory research and is coproducing knowledge and practice [14] by collaborating with the locally based Soliga Adivasi community and district health system actors in Chamarajanagar district in southern India [15].

The research group that forms the Centre has been engaging with the Soliga Adivasi community on Adivasi health for more than a decade [16,17]. Neighbourhood-level disadvantage has come up as a theme during their work and in discussions with the community leaders. The Centre is engaging with the Adivasi communities in co-production of implementation research activities that include running a deaddiction clinic, work on healthcare access, screening and care for sickle cell disease and community-based intervention on sports and mental health.

### Literature search

**Figure 1:**
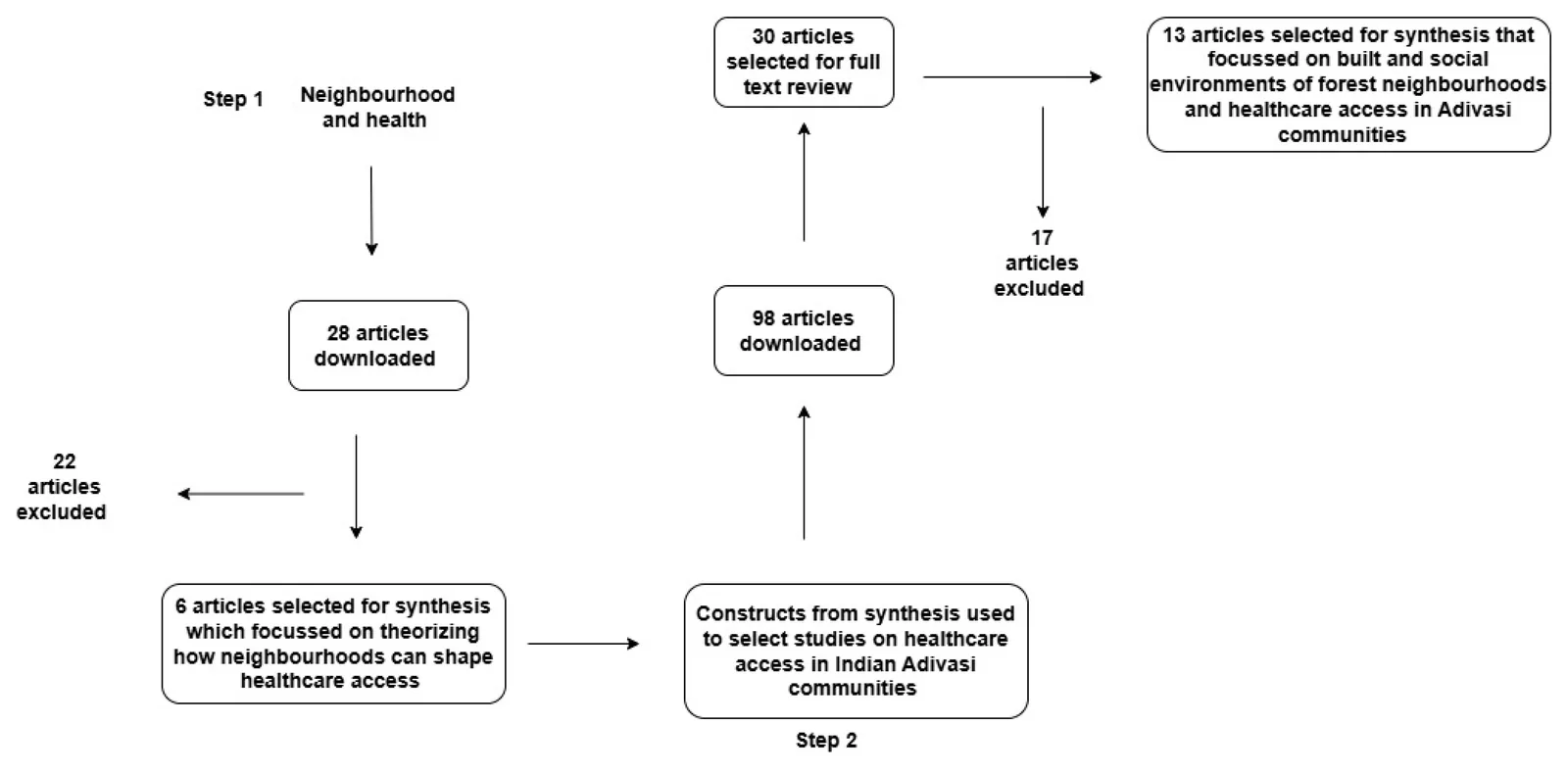
Selection of articles for synthesis.

ProQuest Central, EBSCO host and Google scholar data bases were used to search for studies using the keywords “neighbourhood disadvantage”, “forest rights”, “healthcare access”, “marginality”, “livelihood”, “political ecology”, “tribal”, “indigenous” and “Adivasi”. In step 1 articles related to neighbourhood and health were screened (Figure 1) and six articles were selected using inclusion and exclusion criteria. In step 2 articles were screened using findings from step 1 and final set of thirteen articles were selected for synthesis. A summary of these papers can be found in Table 1.

### Conducting the analysis

**Figure 2:**
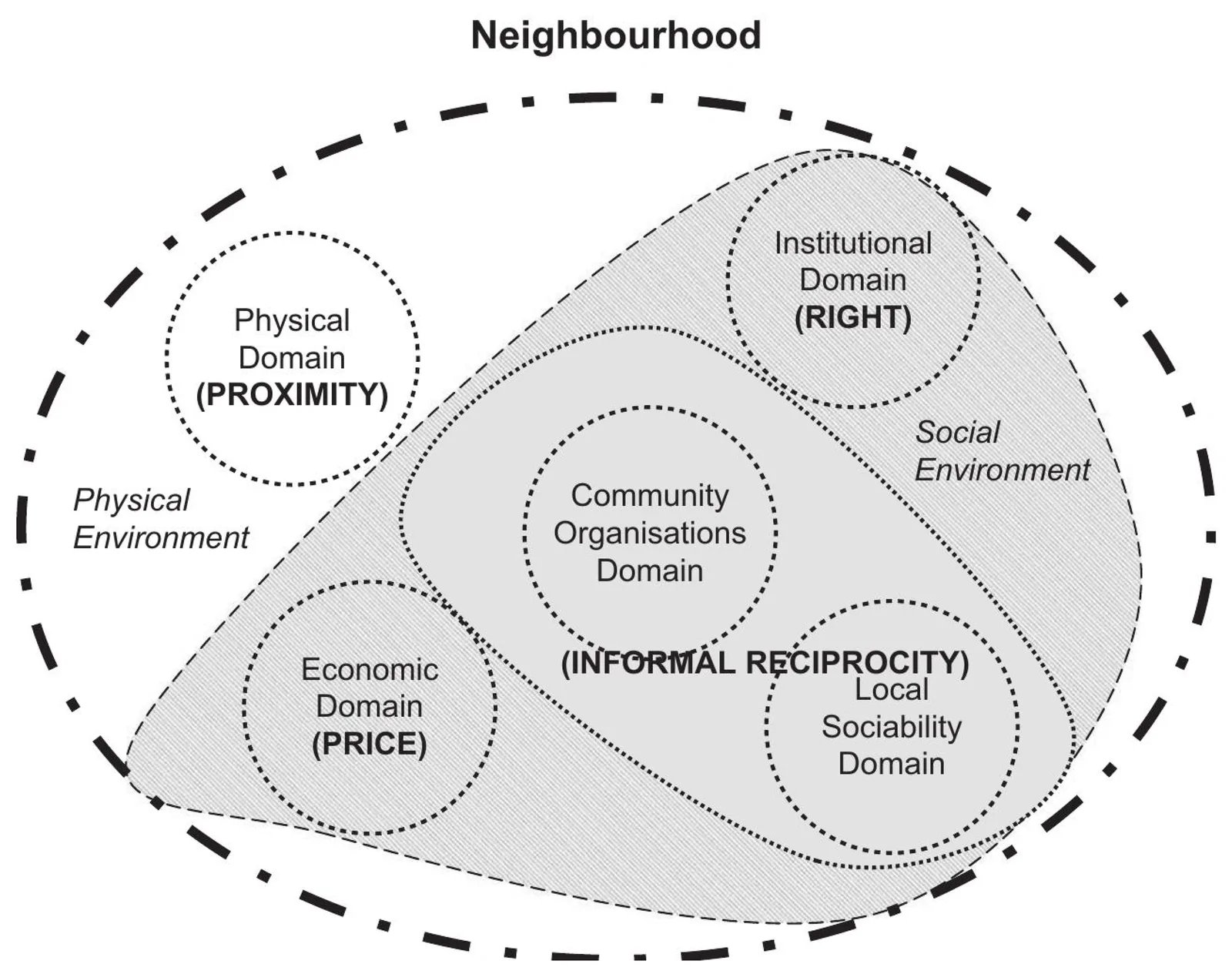
Neighbourhood environments and rules of access as described in “Health inequalities and place: A theoretical conception of neighbourhood” [9].

We selected six papers (Figure 1) to conceptualise healthcare access in relation to neighbourhood environment. These papers helped us understand neighbourhood in terms of built environment, social environment and history of the place. Following this we selected thirteen studies from India which focussed on the built environment, social environment and institutional environment of forest neighbourhoods and healthcare access in Adivasi communities living in forest neighbourhoods. We grouped the thirteen articles into different sets namely Adivasi neighbourhood vulnerability, health systems response, macro-social vulnerability of Adivasis, neighbourhood environment and finally social organisation. This grouping helped us review the articles in relation to history of the place, built environment and the social environment of forest neighbourhoods. We used reciprocal translational analysis as a method in CIS [12] where we first examined the six papers on neighbourhood and health and applied constructs from one study onto another study and so on to conceptualise how health can be understood in relation to neighbourhood. The constructs were then applied to and compared with empirical studies from India on physical and social environments of forest neighbourhoods and healthcare access in forest associated Adivasi communities living in. We specifically used Bernard’s framework (Figure 2) on neighbourhood environment [9] to understand physical and social environments of forest neighbourhoods in India. This framework conceptualises a neighbourhood in terms of its physical and social environment and how access to health-related resources is governed by different rules of access, namely proximity, price, rights and informal reciprocity.

**Figure 3:**
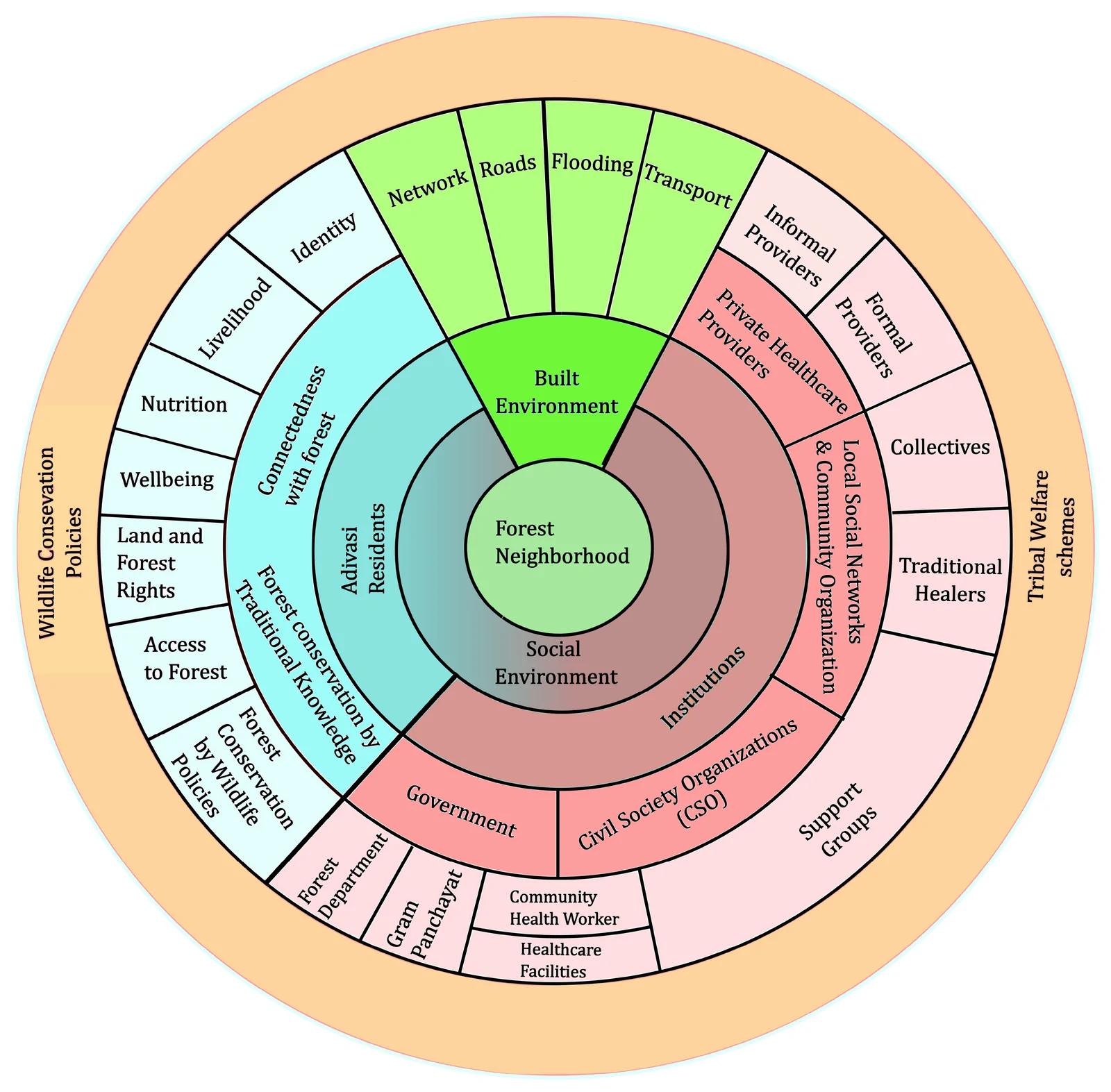
The conceptual framework for built and social environment of a forest neighbourhood in India. Colour legend: built environment (green); social environment (blue and pink); institutional environment (pink) and Adivasi residents (blue).

We have used Bernard’s framework as a base to build our framework and there are some differences which have emerged in our framework. We define the built environment, not only in terms of proximity as given in Bernard’s framework (Figure 3), but also include availability of mobile networks, challenges due to flooding during rains and availability of roads and transport in the built environment. These are critical components that impact access to villages and to areas outside. In our framework in addition to Bernard’s components of the social environment, we added *Connection of Adivasi communities with the forest* as an important component of the social environment. For forest-associated Adivasi communities in India, their lives are deeply connected with the forests in terms of their identity, livelihood, nutrition and knowledge about forests. In the first step we develop the lens of neighbourhood as a physical and social environment and apply it to create a conceptual framework describing physical and social environments of a forest neighbourhood in India. This helped us to understand healthcare access in Adivasi communities in relation to their living environments.

### Positionality

In this paper we examine healthcare access in Indian Adivasi communities from the lens of neighbourhood environment. All authors are non-Adivasi and we acknowledge social power differences between Adivasi and non-Adivasi communities in India. The first author, AJ is a PhD student associated with a broader research group working in Adivasi health [15]. AJ has prior work experience as a clinical trainer and community health researcher in two Adivasi landscapes for three years. The second author, PNS, is one of the principal investigators at the research group and has been involved in collaborative research with the Soliga Adivasi community for more than ten years. AKH is a public health researcher based in Sweden and has experience of working with the rural communities in the mountains of northern Sweden. SG is a public health researcher based in Delhi and works with marginalised communities such as the waste-picking communities in Northern India. Since this is a literature synthesis, ethics approval was not needed as it did not involve collection of any primary data.

## Results

In our findings, we start by explaining our lens of *Neighbourhood as a physical and social environment* for which we selected six review papers that focused on theoretical concepts of health and healthcare in relation to neighbourhoods. These papers helped us understand neighbourhood in terms of its built environment, social environment and how policies can shape the environment. Following this, we present our conceptual framework (Figure 3) created from applying this lens to understand Indian forest neighbourhoods in relation to healthcare access. We select thirteen studies from India, some of which are about the physical and social environments of forest neighbourhoods and some are about healthcare access in Adivasi communities living in forest neighbourhoods. Our framework focuses on three themes which are *Connectedness of lives of Adivasi people with the forest, Built and social environment of forest neighbourhoods* and *Institutional environment of forest neighbourhoods*. These themes are explained in the following sections after introduction of the conceptual framework.

### Neighbourhood as a physical and social environment

A concept paper on neighbourhood and health by Macintyre et al. [6] explains how physical and social environment of a neighbourhood can influence health and access to health-related resources through employment opportunities, educational provision, transportation, housing, recreation facilities, retail provision, and incivilities like vandalism and crime. The same study reports on how social networks and social cohesion, cultural norms and values are some features that influence health outcomes and organisation of healthcare services in a neighbourhood. Kirby and Kaneda in their study on neighbourhood socioeconomic disadvantage report how environmental hazards like air pollution, noise, hazardous waste and industrial effluents can impact physical environments of neighbourhoods [11]. The same study reports that a disadvantaged neighbourhood may lack municipal services such as policing, fire and sanitation and socioeconomic disadvantage in a neighbourhood may impact the physical, service and social environment, creating difficulty in finding, travelling to and affording healthcare services.

Neighbourhood health research in literature is often referred to in terms of composition and context of a place. Composition refers to people living in a place and health outcomes might be similar for people with similar characteristics, for example poor people might have similar death rates, irrespective of the place they live. Whereas context refers to the physical and social environment of a place and gives a place-level explanation. Some studies refer to context and composition as two separate entities of a neighbourhood, while some studies argue that they are not two separate entities and might influence each other [6]. In many quantitative studies on neighbourhood and health, context considered as a confounder or a black box, without availability of theory to explain the pathways between context of a neighbourhood and how it shapes health outcomes of its residents. Traditionally neighbourhood or place in relation to health has been explained using environment agent host model and public sanitation infrastructure. In a concept paper by Chitewere et al., neighbourhood and health is examined using political ecology as a lens where social, political, environmental and economic processes such as land use pattern and land access shape the local environment [18].

### Conceptual framework for built and social environment of a forest neighbourhood in India

In our framework (Figure 3), we conceptualise forest neighbourhood into the built and the social environment. For a forest neighbourhood, availability of mobile networks, condition of roads, flooding of streams during rains and the forms of transport available, form the built environment. We conceptualise the social environment as two different components in this framework, namely connectedness of Adivasi people with the forest and the Institutional environment of the forest neighbourhood. Connectedness of the Adivasi people with the forest is in the form of identity, livelihood, nutrition, well-being, forest rights and the traditional ecological knowledge that they are equipped with from the experience of living in the forest for years. The institutional environment is conceptualised as the different actors located in the region and how they shape access to healthcare. The different actors are the Adivasi people, civil society organisations working on health and healthcare, government healthcare facilities, community health workers, the locally organised collectives of the people, the Gram Panchayat which is the locally elected body and the Forest department. The three themes, one arising from the built environment and two from the social environment are expanded below.

### Built and social environment of forest neighbourhoods in India

This theme is about neighbourhood environment in forest villages in India explaining how local environments shape quality of life of its residents, how the environment itself is affected by external policies and what is the condition of healthcare infrastructure in forest neighbourhoods.

#### Built environment of a forest neighbourhood

Geographic barriers such as roads and availability of transport is a big challenge for forest living Adivasi communities [19]. Wildlife protection and forest conservation rules significantly shape the built environment of the neighbourhood, such as roads, transport and construction activities and also impact access to forests for the Adivasi communities living there. In a study by Nallala [20] based in Odisha, they categorised “hard to reach” areas into reachable, remote and extremely remote. Reachable areas were accessible by concrete roads, whereas remote and extremely remote areas were about two to up to 20 km away, respectively from the motorable road. Extremely remote areas in this study were occupied by particularly vulnerable tribal groups (PVTGs), and electricity and internet were extremely inadequate in these areas. In a study done in central India, a quick evaluation of 700 households drawn from 19 randomly selected Baiga (PVTGs) hamlets, 91% were staying in Kaccha houses, 34% had wired electricity connection and erratic electricity supply and 10% had toilet availability inside homes [21]. In a study carried out in a remote rural district in Jammu and Kashmir India geographic information system was used to map health facilities and their geographic distribution in the area [22]. The study reports that two third of the population remained unserved for a package of services including immunization care, delivery and in-patient care.

#### Social environment of a forest neighbourhood

Chitwere et al. [18] used the lens of political ecology to understand the historical and structural processes that shape the physical and social environment of a neighbourhood. This lens combines cultural ecology referring to people living in a place, their way of life, their physical environment and political economy of a place. In a study carried out in Banswara, a rural Adivasi district in Rajasthan, diarrhoeal infections were studied among children in the context of environmental, economic, socio-cultural and institutional factors [23]. The study recognised the absence of local opportunities to sustain financially secure livelihoods and phenomena such as temporary seasonal migration added stress to families to maintain infant caring practices. In the rural villages of this study, poverty was prevalent, especially in the case of Adivasi people, there was high dependency on government schemes to improve the standard of living and insufficient public financial resources were an obstacle to improve water, sanitation and housing. In another study done in an Adivasi district of Karnataka in southern India, local factors at the village level such as environment, communication, transport, governance and social and familial relationships affected quality of life of its residents [24].

### Connectedness of Adivasi people’s lives with the forest

In a study by Moosan et al. [25] on healthcare access in a tribal community in Kerala, they explain how for forest-based Adivasi communities in India, forest is an integral part of their living environment not only in terms of their livelihood and culture but also as a connecting thread with their ancestors. Kujur et al. [26] in their paper on land alienation among Adivasi communities explain that land is a marker of identity and belonging to a region and the community and their land holding cannot be seen in isolation as it is part of an eco-system involving the forest and its resources. The term Adivasi reflects a collective solidarity to express their belongingness to territories that they have lived in for years. The lack of entitlement to forest resources for forest-based Adivasi communities contributes significantly to their continued poverty. Abel and Frohlich in their article on health inequalities [27] refer to Sen’s capability approach according to which capabilities are people’s ability to do or achieve something which is important to their wellbeing and is valuable to them. This approach is applied to indigenous perspectives by Sangha et al. [28], who argue that indigenous people’s capabilities require including connections with their land along with spiritual and cultural knowledge that is important for their wellbeing. The also explained the existent mutual exchange between ecosystems and indigenous economy, social and cultural worlds. Excluding Adivasi communities from their living spaces can have a direct impact on their well-being and human rights, as well as on sustainable conservation of forests [29].

Kujur et al. explain how alienation of land has disturbed the socio-cultural milieu of forest living Adivasi communities, affecting their identity, livelihood, culture, food security and health. The lack of social and economic capital and their unfamiliarity with competitive job markets have often forced them to turn into casual labourers in the non-agricultural and urban economies [26]. They show that loss of their land rights might gradually wipe out their identity, culture and livelihood. Rai et al. report about the Soliga Adivasi community living inside a tiger reserve in south India who have experienced restricted access to forests due to tiger conservation policies resulting in a high impact on food security, livelihood and ecosystem of the households and the forest [30]. The same study reports that the practice of growing of diverse crops earlier has shifted to monoculture cash crop such as coffee and a ban on hunting has reduced the food basket of the indigenous community. Restrictions on using their land and resources have forced them to replace farming with becoming labourers on nearby coffee estates. In another study from an Adivasi neighbourhood in Kodagu district in south India [24] most inhabitants now work either as owners of plantations, workers or traders in the coffee business and some of them are immersed in the mainstream system occupying marginal positions, selling their workforce to the forest department or to the coffee plantations.

Such precarity in livelihoods can act as a barrier in accessing healthcare services as was reported in a qualitative study [31] to understand barriers to utilize healthcare services among Bodo and Rabha tribes in Udalguri and Baksa districts of Assam. This study reported affordability of services including direct and indirect costs as a barrier, especially for participants who were informal labourers, cultivators and farmers for whom, money was not always available to them. In the Health Access Livelihood Framework [7] livelihood assets are one of the key determinants of illness recognition and treatment seeking behaviour which in turn impact access to healthcare. According to this framework, the livelihood assets comprise of human capital (local knowledge, education, skills), social capital (social networks and affiliations), natural capital (land, water and livestock), physical capital (infrastructure, equipment and means of transport) and financial capital (cash and credit).

### Institutional environment of forest neighbourhoods in India

Many forest villages where Adivasi people live are called Schedule areas which were created under Article 244 of the constitution to preserve tribal autonomy, their culture and economic environment, to ensure social, economic and political justice and to preserve peace and good governance [32]. There are special provisions with the Panchayat extension to Schedule areas act (PESA) with Gram Sabhas or collective meetings of the residents being important for local self-governance [33]. The Wildlife Protection Act 1972 and Forest conservation Act 1982 were brought in to conserve forests and wildlife which is critically important, however it is equally important to consider the human rights of the Adivasi people whose lives are closely interlinked with forests. Several decades of advocacy work by Adivasi social movements and rights groups resulted in the promulgation of Forest rights Act by the Indian parliament. The various rights recognised in this act include right to hold and live in forest land, right to own, collect, use and dispose minor forest produce, right to development facilities such as schools, health centres, roads and more, right to protect conserve and manage community forest resources and right of access to biodiversity and community rights to intellectual property [34].

We have used Bernard’s [9] framework to understand institutional environment of a forest neighbourhood in India. According to this framework, there are four sets of rules that regulate availability and access to health-related resources namely proximity, prices, rights and informal reciprocity [9]. These rules further give rise to five domains that are physical, economic, institutional, local sociability and community organisation domain and these domains cut across the physical and social environment of a neighbourhood. Physical domain is governed by proximity to resources which in turn can be shaped by the interplay of economic forces, institutional arrangements and relations of informal reciprocity. Institutional domain is governed by citizen rights. Informal reciprocity consists of social networks and community action, and it can be a pathway through which people can transform their economic and institutional environment. We find this framework useful for our study as it conceptualises neighbourhood into a physical and social environment and uses different domains such as institutional domain, physical proximity and community organisation to understand availability of and access to health-related resources.

We apply Bernard’s [9] rules of access, to institutions in forest neighbourhoods in India and conceptualise the institutional environment in our framework (Figure 3). To this list, we have added welfare as the logic on which civil society organisations operate. Access to health-related resources and healthcare through the government facilities and community health workers are governed by rights. Healthcare access is also common through informal care providers based within villages who operate on the logic of price, similar to other private for-profit healthcare providers. Civil society organisations operate through logics of welfare, local social networks and sometimes by enabling local community organisation. Informal reciprocity as a rule of access is based on local sociability and community organisation. Local sociability within residents is based on trust and social networks to enable access to health-related resources. Traditional healers are an integral part of care provision based on trust and social networks. Local community organisation can be in the form of collectives formed by people themselves or enabled by civil society organisations. A Gujarat based [35] study on social accountability for maternal health outcomes found that community health workers, the gram panchayat, the village health and sanitation committee and civil society groups formed by community based organisations acted as formal structures for social accountability.

## Discussion

Physical and social environment of a neighbourhood can be shaped by external factors and policies which can often be outside the control of residents of the neighbourhood and we use the concept of ‘Structure’ and ‘Agency’ to understand this. Abel and Frohlich in their article on health inequalities [27] refer to Weber who used the terms structure and agency to explain life chances and life choices respectively, which are shaped by opportunities, resources and the capital available to people and the interaction between them. Structure consists of material aspects and non-material aspects such as normative rules of the community or status group. Non-material aspects can be actors or policies that shape the distribution of resources in the environment and the power relationship between these actors. Agency, on the other hand, is the social choices that are made by people, and these choices operate within limits of social structures. According to Gidden’s structuration theory that is used by Bernard in their framework [9], social structures impose constraints and offer opportunities that shape and orient people’s behaviour and conversely, individuals are agents whose reflexive and routinised practices reproduce and transform social structures.

Forest neighbourhoods in India where Adivasi communities live are contested spaces where there is a mandate for wildlife and forest conservation as well as safeguarding of the cultural identity and human rights of the Adivasi people living in these spaces. The built and social environment of a forest neighbourhood can be seen in terms of structure and agency as mentioned above. There are many external factors such as wildlife conservation policies, administration by the gram panchayat and presence of different actors such as government institutions, civil society organisations, private healthcare providers and locally organised collectives that shape the built and social environment of a forest neighbourhood in relation to healthcare access. The lives of Adivasi people are closely connected with the forest in terms of their identity, livelihood, nutrition and health and all of these are impacted due to their alienation from land or restricted access to forests. Access to healthcare for Adivasi communities is situated within these built and social environments and barriers in healthcare access cannot be seen in isolation from these factors. These form critical components of the social determinants of healthcare access in forest neighbourhoods.

The institutional environment of forest neighbourhoods consists of actors that are governed by different rules of access to health-related resources. The government institutions are governed by welfare and rights of the people and civil society organisations are governed by welfare. Private providers, including formal and informal providers, are governed by price and welfare. There are also locally organised collectives formed in some Adivasi regions to represent their needs and voices with other actors. The forest department is another important actor governing wildlife-related policies in a forest neighbourhood. Presence of these actors and the relationship between them is an important part of the social environment of a neighbourhood. In a study in a tribal community in Kerala [25] collaboration between a primary health centre (PHC) and a local private facility was organised to help with antenatal ultrasounds that were reimbursed using government insurance schemes. As advocated in the health in all policies approach and the sustainable development goal seventeen (SDG 17) [36] which stands for Partnership for goals, health when considered as a shared goal by the diverse set of actors in the neighbourhood can result in better intersectoral planning to improve healthcare access for its residents. Adivasi people have developed traditional ecological knowledge as a result of living in forests for centuries, which is a powerful tool that can contribute to wildlife conservation [29]. A study by Sangha et al. in the Soliga Adivasi community living inside a tiger reserve in southern India demonstrates that faster and better conservation outcomes can be achieved when local people manage forests that they live in with support from government and non-government authorities [37]. Shah et al. in their study propose forest management as a livelihood strategy to address poverty among Adivasi communities, contributing to their socioeconomic development as well as mitigation of climate change by protecting forest ecosystems [38].

### Strengths and limitations

This study examines healthcare access in relation to the local physical and social environment, which is a novel lens bringing in a ‘Place’ perspective in terms of built and social environment. It helps in understanding healthcare access in Adivasi communities in relation to their living environments. Using critical interpretive synthesis as a method has helped in working with literature that is focussing on two different phenomena here, namely neighbourhood environment and access to healthcare and theorise the relationship between them in the form of a conceptual framework.

We acknowledge we have not coproduced knowledge [14] in this review with Adivasi views and authorship. All authors in this study are from non-Adivasi communities. This study is part of a broader research group that is carrying out implementation research activities that involve coproduction of knowledge and practice with the active engagement of Adivasi people as co-researchers [15]. While this synthesis describes the built and social environments of forest neighbourhood in India, it is limited in explaining power relations between the different actors and how that can impact healthcare access. In many forest neighbourhoods there are non-Adivasi communities living as well and their dynamic with the Adivasi communities is an important part of the social environment, though we have not focussed on this aspect in this study. Papers were selected and read by one reviewer who is the first author here, as this is part of her PhD and there is a limitation of a single reviewer at that stage.

In terms of transferability of this study, the findings can be applicable to the living environments of other forest-based Adivasi communities within India, as Connection of the life Adivasi people with the forest is a common phenomenon not only in India but also in indigenous communities across the world. There might be differences across different regions within India in the institutional environment, in terms of the actors the relationship between them and local governance patterns.

### Implications of the study

This paper contributes to academic literature on Adivasi health by using ‘place’ as a lens, which is a new approach to examine healthcare access in forest living Adivasi communities and is particularly relevant for them as their lives are closely connected with their living environment. This paper is based within a larger centre working on Adivasi health that has been engaging with local policy actors. The research team will share the findings with the local policy actors and the local Adivasi communities as well. This literature synthesis is part of a doctoral work and is being followed up by a qualitative inquiry on healthcare access for a forest-associated Adivasi community living in central India.

### Conclusion

It is important to explore neighbourhoods in terms of their physical and social environments to examine the local availability and distribution of public services like education, healthcare and transport and how they are affected by policies and institutions working in the neighbourhood. It is the first step towards working together across different departments to improve access to these services locally. For a forest neighbourhood in rural India, the Adivasi residents and the connection of their lives with the forest is a key component to understand Adivasi health. Alienation or restricted access to forests for Adivasi communities impacts their identity, livelihoods, food security, health and well-being. The built environment of a forest village mainly consists of roads, public transport and related structures and is impacted considerably by wildlife conservation policies and local gram panchayat administration. Access to and navigation through the built environment is critical for access to healthcare within and outside the forest neighbourhood. The social environment of a forest village is formed by the different actors in the neighbourhood namely Adivasi and non-Adivasi residents, government institutions, private institutions, local social networks and locally organised people’s collectives or support groups. Access to health-related resources and healthcare, through these different actors are governed by different rules of access namely proximity, rights, price, welfare and informal reciprocity. Understanding the local physical and social environments of a forest neighbourhood can help in better planning of healthcare services by intersectoral collaboration between the different actors and designing services keeping the life stories of the residents in mind. While this study helps in exploring the local physical and social environment of a forest neighbourhood, more studies from different Adivasi regions within India can help in understanding and documenting different local contexts which might have similarities and differences. Further studies need to be conducted to understand how different actors in the neighbourhood can collaborate towards a shared goal of improving health and healthcare access at the local level.

## Figures and Tables

**Table 1:** Papers used in the synthesis.

SN	Papers	Key message
1	Bernard, P., Charafeddine, R., Frohlich, K. L., Daniel, M., Kestens, Y., & Potvin, L. (2007). Health inequalities and place: a theoretical conception of neighbourhood. Social science & medicine (1982), 65(9), 1839-1852.	This paper conceptualises access to health-related resources in a neighbourhood according to different rules of access, namely proximity, price, rights and informal reciprocity. These rules give rise to five domains which are physical, economic, institutional, local sociability and community organisation. It gives a theoretical explanation of distribution of health inequities at a neighbourhood level. The authors propose that resources and opportunity structures at the neighbourhood level can be a source of inequalities and social forces can shape the physical environment.
	Macintyre, Sally, and Anne Ellaway, ‘Neighborhoods and Health: An Overview’, in Ichiro Kawachi, and Lisa F. Berkman (eds), Neighborhoods and Health, 1st edn (New York, 2003; online edn, Oxford Academic, 1 Sept. 2009), accessed 27 Mar. 2025.	This is a concept paper about neighbourhoods and health. It talks about composition and context in relation to place. Composition refers to people living in a place and focuses on their individual characteristics. Context refers to physical and social characteristics of the place. The chapter argues that traditionally there has been more focus on studying individual behaviours and less focus on studying local environments. It explains how features of the local physical and social environment can shape health such as employment opportunities, educational provisions, transportation, roads and more.
	Ryvicker M. (2018). A Conceptual Framework for Examining Healthcare Access and Navigation: A Behavioral-Ecological Perspective. Social theory & health : STH, 16(3), 224-240.	This paper talks about healthcare navigation in relation to built environment, social environment and healthcare environment. Social environment includes social relationships, perceived social cohesion and support and collective efficacy. Built environment includes roads, highways, public transport.
	Bell, S., Wilson, K., Bissonnette, L., & Shah, T. (2013). Access to Primary Health Care: Does Neighborhood of Residence Matter? Annals of the Association of American Geographers, 103(1), 85-105.	This study considers spatial and aspatial characteristics of populations and physicians to study access to primary healthcare at the neighbourhood level. This study shows that accessibility is considerably reduced for linguistic minorities. Language as an aspatial characteristic could affect individual’s ability to access primary care.
	Chitewere, T., Shim, J. K., Barker, J. C., & Yen, I. H. (2017). How Neighbourhoods Influence Health: Lessons to be learned from the application of political ecology. Health & place, 45, 117-123.	This paper applies political ecology as a lens to understand how neighbourhood can shape health. Neighbourhoods have been traditionally studied in relation to impact of physical and built environment on health outcomes or study of social phenomena through quantitative methods. There is lack of theory and studies examining pathways of how place or neighbourhoods shape health. It proposes political ecology as a lens according to which neighbourhood environments are products of historical, social, political and environmental processes. History of the place, structure which is the physical and social environment of a place and agency of people contribute to the existing environment of a place.
	Kirby, J. B., & Kaneda, T. (2005). Neighborhood socioeconomic disadvantage and access to health care. Journal of health and social behavior, 46(1), 15-31.	This study examines the association between neighbourhood socioeconomic disadvantage and access to healthcare. Socioeconomic disadvantage at the neighbourhood level may give rise to physical, service and social environments that can impact access to healthcare services for residents. Socioeconomic disadvantage at the neighbourhood level can reduce resources available such as churches, schools and voluntary organisations which can act as sources of information, social networks and social support. According to this review, there is lack of empirical studies to explain this association.
	Domínguez, L., & Luoma, C. (2020). Decolonising Conservation Policy: How Colonial Land and Conservation Ideologies Persist and Perpetuate Indigenous Injustices at the Expense of the Environment. Land, 9(3), 65.	This article critiques current conservation approaches that do not acknowledge the rights of the Indigenous people living there and their traditional ecological knowledge. It gives example of four different Indigenous communities in four different countries and how conservation policies have affected their lives.
	John Kujur, Irudaya Rajan S., Udaya S Mishra, Land Vulnerability among Adivasis in India, Land Use Policy, Volume 99, 2020, 105082, ISSN 0264-8377	This paper traces different forms of land alienation among Adivasi communities which is also a global phenomenon. It starts with history of land redistribution in India which was linked to caste hierarchy and the different land reforms in India. Most tribal communities live in rural areas and land is a source of livelihood for many rural communities. Control over land has shifted over time and land use shifted from livelihood to individual property accumulation. This article talks about land reforms as well. Land distribution was also influenced by caste and since Adivasis were outside the caste identity, their land holdings were not affected much. This study maps land vulnerability among Adivasi communities in India. It also refers to theories of land alienation where land can be expropriated for public interest or people can be displaced from their lands by the state. Historically there have been different ways through which Adivasi communities have been dispossessed from their land, through mortgage, marital alliance or bonded labour.
	Boro, B., & Saikia, N. (2020). A qualitative study of the barriers to utilizing healthcare services among the tribal population in Assam. PloS one, 15(10), e0240096.	This study was done in Udalguri and Baksa districts in north east India to understand barriers to healthcare service utilization among the tribal population. Patients and providers both were interviewed. Barriers from the patient’s side in accessing care were affordability, quality of care in the government facilities, poor quality of interpersonal care, lack of medicines and other equipment, more dependence on traditional medicines, distance to health facilities and low level of education and ignorance. Perspectives from the providers side included overburdening of health facilities, lack of education and patient non-compliance, poor transport and communication and lack of government intervention.
	Rai, N.D., Benjaminsen, T.A., Krishnan, S. and Madegowda, C. (2019), Political ecology of tiger conservation in India: Adverse effects of banning customary practices in a protected area. Singapore Journal of Tropical Geography, 40: 124-139.	This paper examines consequences of restricted access to forests by the conservation policies on lives of the Soliga Adivasi people living inside a protected area in India. A ban on customary practices has led to increased weed growth in the forests and restricted access to forests have impacted livelihoods as well as changed food baskets of the Soliga Adivasi people living inside a tiger reserve in southern India.
	Zorondo-Rodríguez, F., Gómez-Baggethun, E., Demps, K., Ariza-Montobbio, P., García, C., & Reyes-García, V. (2014). What Defines Quality of Life? The Gap Between Public Policies and Locally Defined Indicators Among Residents of Kodagu, Karnataka (India). Social Indicators Research, 115(1), 441-456.	This paper compares factors important for quality of life as given in the human development index (HDI) and the factors told by residents of a tribal community in Kodagu district in Karnataka. Human development report is a more top-down indicator and there exists a gap between factors in the report and those narrated by local people that are important for their quality of life. There are some overlapping factors and some gaps between the report. Local environmental factors such as transport, family, social connections agriculture, environment and governance are important factors that affect quality of life of people. The study recommends inclusion of factors important for quality of life as defined by the residents as well while making human development indices.
	Vila-Guilera, J., Parikh, P., Chaturvedi, H., Ciric, L., & Lakhanpaul, M. (2021). Towards transformative WASH: an integrated case study exploring environmental, sociocultural, economic and institutional risk factors contributing to infant enteric infections in rural tribal India. BMC public health, 21(1), 1331.	This study explores environmental, socio-cultural, economic and institutional environment to understand enteric infection drivers in a rural tribal setting in Rajasthan. Their study is grounded in the socio-ecological model and identifies individual, household, community and society level factors in relation to epidemiology of enteric infections. Community level factors involved natural and built environment, local livelihood and employment and local governance.
13	Dash-Kothari-2013-Forest-Rights-in-India.pdf [Internet]. [cited 2024 Apr 6].	This article traces how land alienation has happened for forest living Adivasi communities through wildlife policies and how lakhs of Adivasi people have been evicted from protected areas. It follows up with explaining the details of the Forest rights Act (FRA), how it is a legal and political instrument to help secure rights of forest living Adivasi communities over their ancestral lands. It also explains about the authorities involved in implementation of forest rights and gives examples of different settings in India where FRA has been implemented.
	Hamal, M., de Cock Buning, T., De Brouwere, V., Bardají, A., & Dieleman, M. (2018). How does social accountability contribute to better maternal health outcomes? A qualitative study on perceived changes with government and civil society actors in Gujarat, India. BMC health services research	This study examines social accountability mechanisms related to maternal health in the setting of Gujarat. The links between health facilities, civil society organisations, panchayat and community members is explored for health sector accountability and the mechanisms related to social accountability.
	Moosan, H., Stanley, A., Vijayakumar, K., Jayasree, A. K., Lawrence, T., & Veena, A. (2019). Impediments to Optimal Health-care Utilization of a Particularly Vulnerable Tribal Group in Wayanad: A Qualitative Study. Indian journal of community medicine : official publication of Indian Association of Preventive & Social Medicine	This study examines experiences of healthcare access for a tribal community in Kerala. The findings include collaborations between government and private institutions, need for sociocultural designed care for tribal communities and increasing awareness of recognising urgency of care among communities
16	Sangha, K. K., Le Brocque, A., Costanza, R., & Cadet-James, Y. (2015). Ecosystems and indigenous well-being: An integrated framework. Global Ecology and Conservation, 4, 197-206.	This paper introduces and integrated model of Indigenous well-being. Indigenous well being is determined by their cultural world, economic world and social world and all these interact with each other and each interacts with their natural ecosystem as well.
17	Shah, A. and O.G., S. (2009), Dwindling forest resources and economic vulnerability among tribal communities in a dry/sub-humid region in India. J. Int. Dev., 21: 419-432.	This study proposes forest management as a livelihood strategy to address poverty among Adivasi communities contributing to both their socioeconomic development and mitigation of climate change by protecting forest ecosystems.
18	Sangha, K. K., Madegowda, C., & Balasubramanian, M. (2024). Reshaping conservation incorporating Indigenous perspectives. Global Ecology and Conservation, 54, e03197.	This paper highlights the ecological knowledge among Indigenous people and Local communities across the world (IPLC). It takes the case of the Soliga Adivasi community in south India and how they contribute to conservation by living inside the tiger reserve. The study describes the negative impacts of fortress conservation methods on lives of Indigenous people and argues for the positive role of Indigenous knowledge in contemporary conservation policies.
19	Obrist, B., Iteba, N., Lengeler, C., Makemba, A., Mshana, C., Nathan, R., Alba, S., Dillip, A., Hetzel, M. W., Mayumana, I., Schulze, A., & Mshinda, H. (2007). Access to health care in contexts of livelihood insecurity: a framework for analysis and action. PLoS medicine, 4(10), 1584-1588.	This paper introduces the health access livelihood framework that combines the lens of public health, social sciences and development. It uses three approaches to examine healthcare access, which are health seeking, health services and livelihoods. Healthcare services are distributed according to policies, institutions, organisations and processes which can be outside of individual control. Livelihood assets consist of different forms of capital such as human, social, natural, physical and financial and mobilisation of these assets is often required to enable healthcare access.

## Data Availability

Not applicable.
